# Salmagundi

**Published:** 1887-04

**Authors:** 


### SALMAGUNDI.

EDITED BY E. G. BETTY, D.D.S.

DON QUIXOTE'S Knight Thoughts were not a circumstance compared with the nightmares Dr. Young used to have.

A LITTLE ammonia or borax in the water you wash your hands with, and that water just lukewarm, will keep the skin clean and soft. A little oatmeal mixed with the water will whiten the hands. Many people use glycerine on their hands when they go to bed, wearing gloves to keep the bedding clean; but glycerine makes some skins harsh and red. These people should rub their hands with dry oatmeal and wear gloves in bed. The best preparation for the hands at night is white of egg with a grain of alum dissolved in it. "Roman toilet paste" is merely white of egg, barley-flour, and honey. They say it was used by the Romans in olden time. Any way, it is a first-rate thing; but it is mean, sticky stuff to use and does not do the work any better than oatmeal. The roughest and hardest hands can be made soft and white in a month's time by doctering them a little at bed-time; all the tools you need are a nail-brush, a bottle of ammonia, a box of powdered borax, and a little fine white sand to rub the stains off, or a cut of lemon, which will do even better, for the acid of the lemon will clean anything. Manicures use acids in the shop, but the lemon is quite as good and is not poisonous, while the acids are.—*The Analyst.*

Dr. Frank A. Hunter, of Cincinnati, has at last taken unto himself a wife and will now look at life through a different camera. He's an amateur photographer you know!

Dr. Keely's paper on "Irregularities" published in this issue of the Register is one of the most valuable he has ever given to the profession. He has made their treatment and correction the work of his life, and the vast experience he has gained makes him an authority second to none. He always writes and speaks to the point so that one does not have to drink a gallon of water to get a cup of tea.

The great majority of practitioners very much dislike to undertake the correction of any but simple cases, the more complex necessarily involving a great deal of time, study, and work to accomplish the object in a satisfactory manner. To all such, we would cheerfully recommend a consultation with Dr. Keely, by correspondence and models, or better still, a visit to him at Oxford, O., with the patient; and the reasonable fee such consultation would involve would more than repay itself by the dentist being put upon the right track in the beginning. Dr. Keely should have announced himself as a specialist years ago, so that his professional brethren might avail themselves of his wisdom and mature judgment, and at the same time acknowledge in a substantial manner, the benefit they have received in behalf of some suffering patient.

Gold Solder.—Dr. McKellops recommends that 89 parts of the plate used (be its carat what it may) be alloyed with 7 parts of silver and 4 parts of copper. This will always prove reliable, flow easily, and most nearly preserve the color of the plate. Should it be necessary to solder *over* this solder, reduce it further in the same proportions, *i. e.*, using the solder itself as the gold to be reduced.

### Echoes of The 43rd Meeting M. V. D. A.

The meeting was a most decided success, both in point of attendance, papers read, discussions, exhibition of new appliances, apparatus, etc. From the interest manifested we judge that the meeting of a year hence will excel it in every particular.

Dr. McKellops stood manfully at his post, talked, argued and exhibited appliances with all the gusto of irrepressible youth. May he live long and never fail to put in an appearance at the annual meeting for lo! these many years.

---

Dr. Van Antwerp don't grow thin worth a cent.

---

Dr. Morrison made his first visit for some years past, but expects to be on hand next year loaded to the muzzle with good things to show and talk about.

---

Through a technicality in the resolution providing for a gold medal, none was awarded this year. It is to be hoped that nothing will occur next time to prevent the award, as it looks very much like the parsimony of the American Dental Association in withdrawing its prize upon a certain occasion. The society should provide a fund for an annual prize, to be contested for, by any one within the legitimate practice of dentistry, be he Jew or Gentile, native or foreign.

